# Germline DNA Damage Response Gene Mutations in Localized Prostate Cancer

**DOI:** 10.3390/medicina60010073

**Published:** 2023-12-30

**Authors:** Tomas Januskevicius, Ieva Vaicekauskaite, Rasa Sabaliauskaite, Augustinas Matulevicius, Alvydas Vezelis, Albertas Ulys, Sonata Jarmalaite, Feliksas Jankevicius

**Affiliations:** 1 Clinic of Gastroenterology, Nephro-Urology and Surgery, Institute of Clinical Medicine, Faculty of Medicine, Vilnius University, M. K. Ciurlionio St. 21/27, LT-03101 Vilnius, Lithuania; 2 Laboratory of Genetic Diagnostic, National Cancer Institute, Santariskiu St. 1, LT-08406 Vilnius, Lithuania; 3 Division of Human Genome Research Centre, Institute of Biomedical Sciences, Life Sciences Center, Vilnius University, Sauletekio Ave. 7, LT-10257 Vilnius, Lithuania; 4 Urology Centre, Vilnius University Hospital Santaros Klinikos, Santariskiu St. 2, LT-08661 Vilnius, Lithuania; 5 Oncourology Department, National Cancer Institute, Santariskiu St. 1, LT-08660 Vilnius, Lithuania

**Keywords:** DNA damage response, germline mutation, localized prostate cancer, next-generation sequencing

## Abstract

*Background and Objectives*: Germline DNA damage response (DDR) gene mutations correlate with increased prostate cancer (PCa) risk and a more aggressive form of the disease. DDR mutation testing is recommended for metastatic PCa cases, while eligible information about the mutations’ burden in the early-stage localized PCa is still limited. This study is aimed at the prospective detection of DDR pathway mutations in cases with localized PCa and correlation with clinical, histopathological, and radiological data. A comparison to the previously assessed cohort of the advanced PCa was performed. *Materials and Methods*: Germline DDR gene mutations were assessed prospectively in DNA samples from 139 patients, using a five-gene panel (*BRCA1*, *BRCA2*, *ATM*, *CHEK2*, and *NBN*) targeted next-generation sequencing. *Results*: This study revealed an almost three-fold higher risk of localized PCa among mutation carriers as compared to non-carriers (OR 2.84 and 95% CI: 0.75–20.23, *p* = 0.16). The prevalence of germline DDR gene mutations in PCa cases was 16.8% (18/107) and they were detected only in cases with PI-RADS 4/5 lesions. *BRCA1*/*BRCA2*/*ATM* mutation carriers were 2.6 times more likely to have a higher (>1) cISUP grade group compared to those with a *CHEK2* mutation (*p* = 0.27). However, the number of cISUP > 1-grade patients with a *CHEK2* mutation was significantly higher in advanced PCa than in localized PCa: 66.67% vs. 23.08% (*p* = 0.047). *Conclusions*: The results of our study suggest the potential of genetic screening for selected DDR gene mutations for early identification of cases at risk of aggressive PCa.

## 1. Introduction

Prostate cancer (PCa) is one of the most common problems faced by the male population in the oncology field. Based on the data from the European Cancer Information System, PCa is the most frequently occurring cancer in men; it was responsible for 23.2% of new cancer cases in men in 2020 [1]. Low-risk localized PCa patients may benefit from active surveillance or can be treated by surgery or radiotherapy, usually resulting in complete remission. Intermediate and high-risk localized PCa patients might suffer from disease progression regardless of the primary treatment. In some patients with inherited specific genetic mutations, PCa may manifest in a more severe course, and resistance to conventional treatment develops earlier [2,3]. The transition of next-generation sequencing (NGS) from research to clinical practice revealed new possibilities in the detection of PCa-specific mutations. Due to progress in genomic technologies, mutation status can be verified not only in tumor tissue but also in human body liquids, and it is more widely used in genetic counseling. Blood-based testing of PCa-specific mutations in metastatic PCa patients became increasingly promising, while considering these alterations’ detection for localized PCa is still limited and not widely adopted in clinical practice.

Despite various genetic and epigenetic alterations detectable in PCa, inheritance of PCa risk is mainly attributed to genetic alterations of the DNA damage response (DDR) pathway [4]. This molecular pathway is responsible for the maintenance of the genomic integrity of the cell, and the main players of this pathway are proteins encoded by *BRCA1*, *BRCA2*, *ATM*, *ATR*, *TP53*, *CHEK1*, *CHEK2,* and some other genes. The proteins of the DDR pathway sense DNA damage, induce cell cycle arrest and DNA repair, and protect cells from deleterious genetic alteration accumulation. Inactivation of the DDR pathway leads to genomic instability, uncontrolled cell growth, and malignization. Strong enrichment of mutations in the genes of the DDR pathway is detectable in PCa tissue, especially in metastatic cases [5,6], while a systemic review of the largest PCa studies [7] suggests even higher median prevalence of these mutations in the germline profile of PCa. *BRCA1*, *BRCA2, ATM, CHEK2,* and *NBN* genes are some of the most important alterations in the PCa genetic evaluation process and are frequently included in extensive PCa gene panels.

Most guidelines suggest DDR pathway mutation screening for familial and metastatic PCa, aiming at personalized therapy with poly-ADP ribose polymerase inhibitors (PARPi) [8]. According to the systemic review median prevalence rate for germline DDR gene mutations in general (unselected) PCa is higher than in the metastatic disease (18.6% vs 11.6%), suggesting a higher burden of these mutations than expected [7]. However, quite a few studies analyzed the DDR gene mutation rate in localized or locally advanced PCa and detected a prevalence of 1.44–9.5% [9,10,11,12,13]. In PCa, germline DDR mutations seem to have a higher prevalence than somatic ones and, due to the low penetrance of some mutations, remain undetected in families until manifestation in aggressive forms of cancer [7]. It was shown that patients with inherited pathogenic mutations of several DDR genes are at increased risk of developing more aggressive forms of the disease, while *BRCA2* mutations are directly associated with poor survival in metastatic PCa [14,15,16]. Metastatic PCa patients with mutant DDR genes already benefit from targeted therapies with PARPi, while patients with the localized disease could be evaluated for the risk of early recurrence or take advantage of personalized treatment options in the future; however, such observations need further investigation. Early detection of DDR mutation carriers is also vital for family members’ consultation due to the high risk of aggressive breast, ovarian, and some other tumors [17]. Since at least a quarter of PCa patients identified with germline mutations lack a cancer-related family history, and the mutations possibly evolve de novo [18], it is important to develop algorithms for the meaningful selection of PCa patients for germline mutation testing.

Our prospective, single-center cohort study aimed to assess the prevalence of germline DDR gene mutations (*BRCA1*, *BRCA2*, *ATM*, *CHEK2*, and *NBN*) in patients with localized PCa that was diagnosed based on positive findings of multiparametric magnetic resonance and ultrasound imaging (mpMRI/UG) fusion-guided targeted biopsy. Associations between mutation status and clinical, histopathological, and radiological data were analyzed and a comparison to the previously assessed advanced PCa cohort [19] was performed.

## 2. Materials and Methods

### 2.1. Patient Cohort

Between 2019 and 2023, a total of 150 patients with suspected PCa were enrolled in this study at the National Cancer Institute (Vilnius, Lithuania). Prostate mpMRI was performed on all study patients and was reported using the Prostate Imaging Reporting & Data System version 2.1 (PI-RADSv2.1) [20]. A positive mpMRI scan was characterized by the presence of PI-RADS lesions with a score ≥ 3. Patients with a positive prostate mpMRI scan, in accordance with their medical history, clinical data, and/or elevated (>3.0 ng/mL) prostate-specific antigen (PSA) level, underwent mpMRI/US fusion-guided targeted biopsy. PSA density was defined as total PSA (ng/mL) divided by mpMRI calculated prostate volume (mL). Biopsy results were evaluated based on the International Society of Urological Pathology (cISUP) grading system. Low-risk localized, intermediate-risk localized, and high-risk localized/locally advanced diseases were defined by the European Association of Urology (EAU) risk stratification groups for biochemical recurrence [21]. This study was performed in line with the principles of the Declaration of Helsinki. This study was approved by the Regional Bioethics Committee (05.11.2019/No: 2019/11-1166-654) and written informed consent was obtained from all participants.

### 2.2. Sample Collection and NGS

Blood samples were collected prospectively into EDTA blood collection tubes according to the standardized clinical procedures. The collected buffy coat was frozen and stored at a temperature of −80 °C. The DNA extraction was performed by using a GeneJET Genomic DNA Purification kit (Thermo Fisher Scientific, Vilnius, Lithuania), following the manufacturer’s instructions. DNA concentration and purity were determined using the NanoDrop 2000 spectrophotometer (Thermo Scientific, Wilmington, DE, USA) as well as Qubit™ dsDNA BR Assay Kit on a Qubit™ 2.0 Fluorimeter (Invitrogen, TFS, Eugene, OR, USA) and stored at −20 °C until use. Targeted DNA sequencing was performed on the Ion Torrent™ Ion S5™ system, and for the library preparation, Ion AmpliSeq™ Library Kit 2.0 and custom On-Demand Panel (consisting of *BRCA1*, *BRCA2*, *CHEK2*, *ATM*, and *NBN* genes) (from Life Technologies (LT), Carlsbad, CA, USA) were used under conditions provided by the manufacturer’s protocol. Sequencing results were analyzed in the Ion ReporterTM Software (version 5.20.2.0) system (Life Technologies, Carlsbad, CA, USA), verified manually in the Integrative Genomics Viewer (IGV, version 2.6.3) tool (Broad Institute, Cambridge, MA, USA), and compared to the hg19 reference human genome sequence. The pathogenic and likely pathogenic mutations were confirmed if the mutation was listed in the clinical variant base ClinVar, as well as visualized on the Integrative Genomics Viewer 2.4.8 tool.

### 2.3. Statistical Analysis

Statistical analysis was performed using R Version 4.1.1 on R Studio version 2022.07.0 (R Core Team, Vienna, Austria). Oncoprint was created using ComplexHeatmap R package version 2.11.1 [22]. Mutation associations with clinical data were assessed by Fisher exact test, Chi-square test, or *t*-test where appropriate. The odds ratio (OR) was computed by analyzing two-by-two tables. The statistical significance of the OR was evaluated using Fisher exact tests. Results were considered statistically significant if the *p*-value was <0.05.

## 3. Results

### 3.1. Characteristics of Study Group

In a study cohort of 150 patients, 11 cases were excluded from further analysis due to clinical or sample quantity reasons. After mpMRI/US fusion-guided targeted biopsy, all patients (*n* = 139) were divided into two groups: 107 were histologically confirmed with localized PCa, and 32 patients without PCa diagnosis were assigned to the control group. Based on the presence or absence of pathogenic mutation in the analyzed genes, the patients were divided into mutation-positive–DDR(+) and mutation-negative–DDR(−) groups.

PCa patients (*n* = 107) revealed PI-RADS lesions ranging from 3 to 5, with a dominance of PI-RADS 4 lesions (67/107, 62.62%). Also, different cISUP grade groups were observed: the most prevalent cISUP grade group was 1 (68/107, 63.55%), followed by grade group 2 (24/107, 22.43%), grade group 3 (11/107, 10.28%), and grade group 4 (4/107, 3.74%). According to EAU risk groups [21], patients with PCa revealed low-risk disease as the most common: 58/107, 54.20%. Intermediate- and high-risk diseases were detected in 44 (41.12%) and 5 (4.68%) patients, respectively. The main clinicopathological characteristics and mpMRI features of PCa patients are shown in Table 1.

### 3.2. DDR Gene Mutations Rates

**Figure 1:**
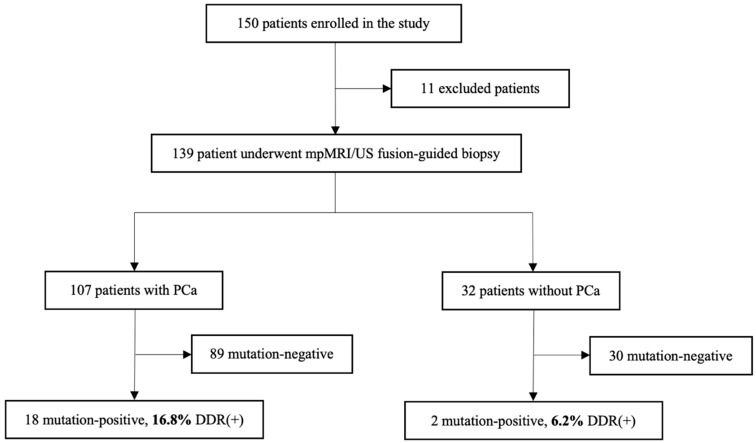
Flowchart of the patient cohort included in the study. Abbreviations: mpMRI—multiparametric magnetic resonance imaging; US—ultrasound; DDR—DNA damage response; PCa—prostate cancer.

**Figure 2:**
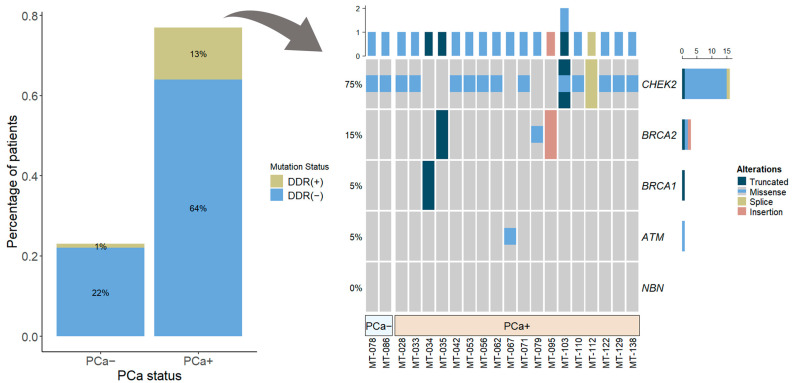
Percentage of patients (*n* = 107) with and without DDR gene mutations: overall (barplot) and according to each selected gene (oncoprint). Abbreviations: DDR—DNA damage response; PCa+—prostate cancer, PCa−—controls.

Out of 139 cases, 14.4% (*n* = 20) were identified with germline mutations of selected DDR genes (*BRCA1*, *BRCA2*, *ATM*, *CHEK2*, and *NBN*) in the blood cells (Figure 1). The mutation rate in the group of cases with PCa diagnosis was 16.8% (18/107), and 19 alterations in DDR genes were found, with one case showing multiple alterations in the *CHEK2* gene: *CHEK2 c.470T>C* and *CHEK2 c.1100delC* (Figure 2). *BRCA1* mutation was detected in one PCa patient (0.93%), *BRCA2*—3 (2.8%), *ATM*—1 (0.93%), and *CHEK2*—13 (12.15%). None of the cases were identified with *NBN* mutation.

In the control group of 32 cases without confirmed PCa diagnosis, only two *CHEK2* gene mutations were detected (2/32; 6.2%). Comparison of localized PCa to control group revealed an almost three-fold higher risk of localized PCa among DDR gene mutation carriers as compared to non-carriers (OR 2.84 and 95% CI: 0.75–20.23, *p* = 0.16), and *CHEK2* mutation was responsible for the doubling in risk of localized PCa (OR 1.95, 95% CI: 0.49–14.18, *p* = 0.51). Due to the small number of cases with DDR gene mutations, the OR comparison did not reach statistical significance.

Comparison to the advanced PCa cohort from our previous study [19] revealed a higher rate of the DDR gene (*BRCA1/BRCA2/ATM/CHEK2*) mutations in the localized PCa than in the advanced disease (16.8% vs. 14.8%, *p* = 0.70).

### 3.3. Clinical Characteristics of DDR Mutation-Positive PCa

Analysis in the localized PCa group (*n* = 107) revealed that the DDR(+) cases (*n* = 18) were younger compared to DDR(−) cases (61.56 vs. 63.69 years, *p* = 0.27) and were presented with slightly lower PSA concentration and PSA density (Table 1). However, prostate volume was higher in the DDR(+) PCa group (55.77 vs. 44.16, *p* = 0.06) (Table 1). Importantly, *BRCA1*/*BRCA2*/*ATM* mutation carriers were markedly younger in the localized PCa cohort than in the advanced PCa group from our previous study [19]: 61.20 vs. 68.30 years; *p* = 0.06.

The localized PCa cases with DDR gene mutations were most frequently identified with PI-RADS 4 (12/18, 66.66%) lesions (Table 1), and the combined occurrence of any DDR gene mutation in PI-RADS 4/5 lesions reached 17.47%. The highest mutation rate was detected in cISUP grade group 4 and accounted for 25.00%. *BRCA1*/*BRCA2*/*ATM* mutation carriers (*n* = 5) revealed higher cISUP grade group scores (cISUP > 1 vs. cISUP = 1) compared to those with *CHEK2* mutation (*n* = 13) and the overall group of patients with mutations: (*n* = 18)—60.00% vs. 23.08%, *p* = 0.27 and 60.00% vs. 33.33%, *p* = 0.34, respectively. There were no statistically significant differences in mutation frequency according to risk groups divided by low-risk and intermediate/high-risk disease (Table 1).

**Figure 3:**
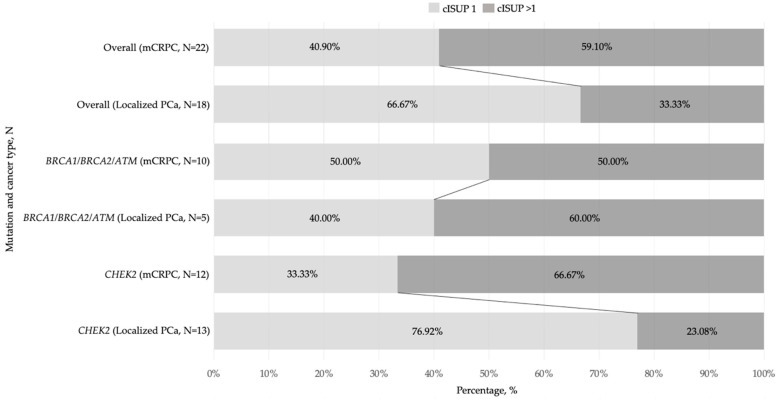
Bar graphs showing a prevalence of various DDR(+) mutations by cISUP grade group in localized and advanced prostate cancer groups, N. Abbreviations: cISUP—International Society of Urological Pathology grade group; mCRPC—metastatic castration-resistant prostate cancer; PCa—prostate cancer.

The DDR gene mutations rate was also high in the cISUP > 1-grade group scores among advanced PCa cases [19] and exceeded mutation prevalence in the localized cISUP > 1 disease (59.10% vs. 33.33%, *p* = 0.13), and the number of cISUP > 1-grade group patients with *CHEK2* mutation was markedly higher in mCRPC as compared to localized PCa: 66.67% vs. 23.08% (*p* = 0.047) (Figure 3).

## 4. Discussion

Currently, PCa in the localized setting is characterized by its heterogeneous nature, whereas standard treatment options are generally well-established and approved by various guidelines worldwide. Nonetheless, in this PCa stage, there remains a significant knowledge gap regarding the significance of germline DDR pathway mutations in the management of the disease. Until the development of castration resistance or the diagnosis of distant-spread diseases, the range of follow-up means or specific treatment possibilities for patients with the alterations remains limited, and the weight of various mutations on the disease aggressiveness remains obscure.

In this study, prospective five DDR gene mutation testing was performed in 139 men who underwent mpMRI/US fusion-guided targeted prostate biopsy. We detected a substantial rate of genetic alterations in histologically confirmed PCa patients, reaching a prevalence of 16.8% (18/107). In comparison, our previous study on advanced castration-resistant PCa (mCRPC) revealed a germline mutation prevalence of 14.8% in the same genes (*BRCA1*, *BRCA2*, *ATM*, and *CHEK2*) [19]. Our data support observations of previous studies [9,10,11,12,13], i.e., that mutation rates can be relatively high in localized PCa in comparison with metastatic disease. This observation suggests that DDR gene mutations are probably early events in the evolution of aggressive prostate tumors and emphasizes the significance of conducting germline testing from the early stages of PCa.

To our knowledge, limited data exist on the association of mpMRI lesions and the presence of DDR gene mutations in localized PCa studies, usually aiming at a radiological nodal status. When analyzing the prevalence of the DDR gene mutations based on the PI-RADSv2.1 scoring system, we found that the combined occurrence of any DDR gene mutation in PI-RADS 4/5 lesions reached 17.47%. Meanwhile, no DDR gene mutations were detected in cases with lower-category (PI-RADS ≤ 3) lesions. Although these results are arguable because of their clinical application, their potential benefit might be seen in the future, when different strategies of germline testing can be adopted in patients with suspicious PCa.

In our study, the DDR pathway mutations were highly specific to cases, and only two controls without confirmed PCa were identified with *CHEK2* gene mutations. In our cohort, DDR gene mutations were associated with an almost three-fold, while *CHEK2* mutations were associated with a two-fold, increase in localized PCa risk. Positive DDR mutation status may not only help to identify PCa but it also is associated with an aggressive course [14,15] and can help verify localized PCa cases that demand timely treatment.

In our cohort, *CHEK2* alteration was found to be the most prevalent (12.15%) in localized PCa, and missense alteration *c.470T>C* was the predominant type of mutation (12/19). Most notably, Wang, Y. et al. [23], revealed that *CHEK2 c.470T>C* significantly increased the PCa risk: OR 1.80, 95% CI: 1.51–2.14, *p* < 0.0001. The significant association between *CHEK2* mutations and PCa risk (OR 1.9, 95% CI: 1.6–2.2, *p* < 0.0001) was also found in a study by Cybulski, C. et al. [24]. Specifically, they observed that truncating *CHEK2* variants was associated with higher risk when compared to missense mutations such as *c.470T>C* [24]. Our study results, in comparison with our previous study [19], revealed a predominance of *CHEK2* mutations in high cISUP grade mCRPC, but not in localized PCa (*p* = 0.047). This encourages further studies of the impact of various *CHEK2* mutation types on the aggressiveness of PCa.

The combination of alterations in *BRCA1*/*BRCA2* and *ATM* genes is associated with more aggressive PCa and is widely investigated in extensive mCRPC trials with PARPi [25,26,27]. In our cohort, *BRCA1*/*BRCA2* and *ATM* mutations accounted for a percentage of 4.67. The prevalence of *BRCA1*/*BRCA2* and *ATM* mutations was found to be greater than the reported rates of low-risk localized PCa patients by Na, R. et al. [9], i.e., 1.44%, and is consistent with the findings in the cohort analyzed by Marshall, C.H. et al. [10]—5.4%. When comparing these alterations individually, with the European ancestry patients from the large study by Lee, D.J. et al. [13], we identified higher mutation rates in *BRCA1* (0.93% vs. 0.77%), *BRCA2* (2.8% vs. 1.0%), and *ATM* (0.93% vs. 0.51%). The Cancer Genome Atlas (TCGA) [5] analysis of 333 primary prostate tumors revealed *BRCA2* and *BRCA1* mutation rates that were quite similar to our study (3% and 1%, respectively), while *ATM* mutations were slightly more frequent than in our study (4% in TCGA vs. 1% in our study). Looking closer at this study on cBioPortal [28], when excluding somatic mutations in this particular cohort, the *BRCA1* mutation rate was 1% and the *BRCA2* was 1.5%, and there were no germline mutations noted in any of the other six genes (*ATM*, *NBN*, and *CHEK2* from our study, and *CDK12*, *FANCD2*, and *RAD51c* from the TCGA study).

In our study, the DDR pathway alterations were less common in low-risk PCa patients than in intermediate- or high-risk diseases compared to non-carriers. DDR gene mutation carriers with *BRCA1*/*BRCA2* and *ATM* alterations were 2.6 and 1.8 times more likely to have a higher (>1) cISUP grade group, compared to those with *CHEK2* mutation and with all mutated cases, respectively. Taken together, our data and the data of other authors [2,3,14,15] suggest that these DDR gene mutations can significantly contribute to a more aggressive course of PCa.

There are several limitations of this study. Although our findings revealed a high percentage of DDR gene mutation carriers among localized PCa patients, the sample size with DDR mutations remains relatively small and reduces the statistical power of the study. Also, only five genes were included in the analysis, and the *NBN* mutation was not detected at all. Several other DDR pathway genes are associated with genetic risk of PCa and may also be important in localized disease. In our analysis, we did not include the family cancer history of the study patients, because family histories were incomplete or inaccurate in a majority of the cases. In addition, we did not investigate how the mutation status may affect clinical outcomes, though this facet merits further studies.

The high prevalence of DDR alterations in localized PCa observed in our and other studies suggests that these mutations are an early event in prostate carcinogenesis. Since DDR deficiency may be associated with an aggressive cancer phenotype, knowledge of DDR gene status in localized stage disease may be critical, requiring more accurate decision-making in various clinical settings, such as choosing active surveillance over radical therapy or surgery over radiation, assessing the optimal timing of salvage radiotherapy after radical prostatectomy, and many other. In addition, DDR gene mutation testing in early-stage localized PCa can provide a better understanding of the molecular biology of PCa and optimize genetic testing strategies for familial cancer management. The impact on clinicopathological data and the correlation between mpMRI findings and mutation status revealed in our study provides additional arguments for wider DDR gene mutation testing in PCa.

## 5. Conclusions

Results of our study display a quite high rate of germline DDR mutations in localized PCa with certain implications on clinical outcomes suggesting a potential benefit of targeted genetic testing for the early identification of mutation carriers at risk of aggressive PCa.

## Figures and Tables

**Table 1:** Clinicopathological characteristics and mpMRI features of localized PCa patients.

Variable	DDR(+), *n* = 18	DDR(−), *n* = 89	*p* Value
Age at PCa diagnosis, years (mean ± SD)	61.56 (±5.66)	63.69 (±7.70)	0.268
PSA level at PCa diagnosis, ng/mL (median (IQR))	5.79 (4.31)	5.90 (3.73)	0.739
Prostate volume, mL (median (IQR))	55.77 (50.57)	44.16 (20.63)	0.057
PSA density (median (IQR))	0.11 (0.07)	0.14 (0.12)	0.170
cISUP grade group			
<3, *n* (%)	15 (83.33%)	77 (86.62%)	0.714
≥3, *n* (%)	3 (16.67%)	12 (13.48%)	
PI-RADS category based on the prostate mpMRI			
PI-RADS 3, *n* (%)	0 (0.00%)	4 (4.50%)	1.000
PI-RADS 4, *n* (%)	12 (66.66%)	55 (61.79%)	
PI-RADS 5, *n* (%)	6 (33.34%)	30 (33.70%)	
EAU risk groups			
Low-risk PCa, *n* (%)	9 (50.00%)	49 (55.06%)	0.694
Intermediate- or high-risk PCa, *n* (%)	9 (50.00%)	40 (44.94%)	

Abbreviations: PCa—prostate cancer; PSA—prostate-specific antigen; cISUP—International Society of Urological Pathology grade group; mpMRI—multiparametric magnetic resonance imaging; PI-RADS—Prostate Imaging Reporting & Data System; EAU—European Association of Urology; SD—standard deviation; IQR—interquartile range.

## Data Availability

The data are not publicly available due to privacy or ethical restrictions.
